# Prevalence of transmitted HIV-1 antiretroviral resistance among patients initiating antiretroviral therapy in Brazil: a surveillance study using dried blood spots

**DOI:** 10.7448/IAS.17.1.19042

**Published:** 2014-09-22

**Authors:** Celina M P de Moraes Soares, Tania R C Vergara, Carlos Brites, Jose D U Brito, Gorki Grinberg, Marcos M Caseiro, Carlos Correa, Theodoro A Suffert, Flavio R Pereira, Michelle Camargo, Luiz M Janini, Shirley Komninakis, Maria C A Sucupira, Ricardo S Diaz

**Affiliations:** 1 Infectious Diseases Unit, Federal University of São Paulo (UNIFESP), São Paulo, Brazil; 2 Virology Research Laboratory, Federal University of Bahia, Salvador, Brazil; 3 Federal District Hospital Foundation, Brasília, Brazil; 4 Molecular Biology Laboratory, Lusíada University, Santos, SP, Brazil; 5 Municipality of Itajaí, Itajaí, Brazil; 6 Municipality of Porto Alegre, Porto Alegre, Brazil; 7 Infection Disease Division, State University of Amazonas, Manaus, Brazil

**Keywords:** transmitted drug resistance, dried blood spots, Brazil, genotyping, antiretroviral therapy, HIV subtype

## Abstract

**Introduction:**

In Brazil, the use of antiretrovirals is widespread: more than 260,000 individuals are currently undergoing treatment. We conducted a survey targeting antiretroviral-naïve individuals who were initiating antiretroviral therapy (ART) according to local guidelines. This survey covered five Brazilian regions.

**Methods:**

The HIV Threshold Survey methodology (HIV-THS) of the World Health Organization was utilized, and subjects were selected from seven highly populated cities representative of all Brazilian macro-regions. Dried blood spots (DBS) were collected on SS903 collection cards and were transported by regular mail at room temperature to a single central laboratory for genotyping.

**Results:**

We analysed samples from 329 individuals initiating highly active antiretroviral therapy (HAART), 39 (11.8%) of whom were harbouring transmitted drug resistance (TDR). The mean CD4+ T cell count was 253 cells/µL, and the mean viral load was 142,044 copies/mL. The regional prevalence of resistance was 17.0% in the Northeast, 12.8% in the Southeast, 10.6% in the Central region, 8.5% in the North and 8.5% in the South. The inhibitor-specific TDR prevalence was 6.9% for nucleoside reverse transcriptase inhibitors, 4.9% for non-nucleoside reverse transcriptase inhibitors and 3.9% for protease inhibitors; 3.6% of individuals presented resistance to more than one class of inhibitors. Overall, there were trends towards higher prevalences of subtype C towards the South and subtype F towards the North. Of the DBS samples collected, 9.3% failed to provide reliable results.

**Discussion:**

We identified variable TDR prevalence, ranging from intermediate to high levels, among individuals in whom HIV disease progressed, thus implying that resistance testing before initiating ART could be effective in Brazil. Our results also indicate that the use of DBS might be especially valuable for providing access to testing in resource-limited and remote settings.

## Introduction

As of December 2012, more than 260,000 individuals were receiving antiretroviral treatment in Brazil, accounting for almost two-thirds of the individuals living with HIV who are followed by the Brazilian Public Health System (www.aids.gov.br). Brazil also provides a network of laboratories offering monitoring tests. However, according to current local guidelines, only patients under virologic antiretroviral failure are entitled to obtain the genotyping tests, which are performed in a network of specialized laboratories [1].

From April 2010 to December 2012, Brazilian guidelines recommended treatment for patients with CD4+ T cell counts below 350 cells/µL. Therefore, we decided to conduct surveys in targeted Brazilian macro-regions of antiretroviral-naïve individuals living with HIV who were initiating antiretroviral treatment according to the local guidelines. We hypothesized that individuals initiating treatment would generally have low CD4+ T cell counts, indicating that HIV disease had progressed, and that those individuals had been infected for long periods of time (at a time when antiretroviral treatment was not widely used and transmitted drug resistance, or TDR, would therefore be rare).

In Brazil, genotype testing is currently offered only to persons experiencing virologic failure. Nonetheless, TDR has been shown to be a pressing issue in Brazil [2]. Furthermore, it is known that multiple subtypes of HIV infection co-circulate in Brazil [3–5], and it is not well understood whether TDR varies among different subtypes. Therefore, we sought to conduct a TDR survey spanning multiple regions of Brazil.

Many surveys for determining the prevalence of HIV TDR have been performed among acutely or recently infected individuals to mitigate the loss of testing sensitivity related to the emergence of wild-type strains. However, in contrast to what is seen in secondary resistance, besides M184V mutation, the replacement of resistant strains with wild-type strains is not likely to occur [6–8], as there is a genetic bottleneck during primary HIV infection that leads to the clonal transmission of HIV of a certain genetic profile [9]. Therefore, we believe that even at a time long after the transmission event, genotype testing would be suitably sensitive for detecting TDR.

## Methods

The HIV Threshold Survey methodology (HIV-THS) of the World Health Organization was utilized in this study. This method considers a low prevalence of TDR to be less than 5%, an intermediate prevalence to be between 5 and 15% and a high prevalence to be levels above 15%. We selected one highly populated city from each of the five Brazilian macro-regions and analysed 47 samples from each city: Manaus (North region), Salvador (Northeast region), Brasília (Central region), Rio de Janeiro (Southeast region) and Porto Alegre (South region). We also included two additional cities that have the highest incidences of AIDS and HIV mortality in Brazil (www.aids.gov.br): Itajaí from the South region and Santos from the Southeast region. The latter was also previously recognized as having high prevalences of TDR, CRF_28 and CRF_29 [10, 11].

When antiretroviral treatment was prescribed, the attending physician invited patients to sign informed consent forms and to provide pre-treatment samples for HIV-1 genotyping as part of this study. Dried blood spots (DBS) were used in this study. An attending nurse or physician collected the samples on SS903 collection cards after a needle puncture of the finger, and then sent them by regular mail at room temperature to a single central lab, the Retrovirology Lab of the Federal University of São Paulo, Brazil, for genotyping. Samples were collected from July 2009 to December 2010.

Genotyping was performed from HIV proviral DNA, which was extracted using a QIAamp Blood Kit (Qiagen Inc., Chatsworth, CA, USA). Protease and reverse transcriptase (RT) regions of the *pol* gene were amplified and sequenced as previously described [11].

TDR was evaluated according to an algorithm from the WHO (updated in 2009) that excludes common polymorphisms and considers 93 mutations: 34 nucleoside reverse transcriptase inhibitor (NRTI) resistance mutations at 15 RT positions, 19 non-nucleoside reverse transcriptase inhibitor (NNRTI) resistance mutations at 10 RT positions and 40 protease inhibitor (PI) resistance mutations at 18 protease positions [12]. Phylogenetic analysis was performed for subtype assignment, in which sequences were aligned to the reference data set from the Los Alamos database using BioEdit version 7.2.3 [13]. For each alignment, phylogenetic analyses were performed using the PHYLIP programme package, version 3.57 [14]. The DNAdist programme was used to calculate distance matrixes based on the maximum-likelihood model, and neighbour-joining trees were generated using the Neighbor and Consense programmes. Statistical significance was assessed with bootstrap tests in a total of 100 replications. Alternatively, phylogenetic analyses were conducted using MEGA software, version 5.2.2 [15].

We analysed predictors of TDR including gender, age, risk factors for HIV acquisition (men who have sex with men, heterosexual exposure, injectable drug use and transfusion before the availability of anti-HIV enzyme immunoassay), reported partner using antiretrovirals and HIV subtype using chi-square and Fisher's exact test.

## Results

DBS specimens were collected in a total of 352 patients. Of these, we were able to amplify nucleic acid sequences in 329 patients. Sample collection was then stopped as 329 was the target number of genotyping tests planned for by the threshold survey method. The prevalence of non-amplifiable sequence was similar across all sites (data not shown).

Overall, the prevalence of TDR was 11.6%. This varied by geographic region (Table 1), ranging from 4.4% in Itajaí to 17.0% in Salvador and Santos. Overall, 6.9% of genotypes showed one or more NRTI mutations, 4.9% had one or more NNRTI mutations and 3.9% had one or more PI mutations. Two- or three-class resistance was 3.6% (1.8% to NRTI and NNRTI, 1.5% to NNRTI and PI and 0.3% to NNRTI and PI). There was one subject with three-class resistance. Specific mutations are described in Table 2. There were no relationships between TDR prevalence and gender, HIV subtype or risk factors for HIV acquisition. Of patients who reported a sexual partner using antiretrovirals, 11.1% exhibited TDR, compared to 23% of individuals who did not know the HIV status of sexual partners (Fisher's exact test *p*=0.06).

**Table 1:** Demographic, virologic and immunological characteristics of individuals according to the different Brazilian regions and TDR prevalence

City/region	Mean age (variation)	Males (%)	Mean CD4	Mean VL log_10_	TDR (%)	2 classes resistance (%)	3 classes resistance (%)
Manaus/N	36 (18–61)	59.6	320 (91–781)	4.6 (2.4–>5.7)	8.5	2.1	
Salvador/NE	38 (19–58)	60.9	227 (3–581)	5.2 (3.7–5.9)	17.0	4.3	
Brasilia/MW	40 (21–63)	77.1	235 (24–644)	5.3 (2.0–6.3)	10.6	4.3	
Rio de Janeiro/SE	41 (27–76)	89.4	233 (25–447)	5.2 (3.5–5.9)	12.8	4.3	
Porto Alegre/S	41 (18–65)	63.3	249 (35–759)	5.1 (3.0–>5.7)	12.2	2.0	
Santos/SE	40 (26–69)	65.7	285 (11–861)	4.9 (2.7–5.5)	17.0	3.7	1.9
Itajaí/S	39 (25–63)	65.9	247 (15–475)	5.2 (2.4–6.2)	4.4	2.2	
Total	39 (18–76)	69.0	253 (3–861)	5.2 (2.0–6.3)	11.6	3.3	0.3

Forty-seven samples have been collected for each site.

**Table 2:** TDR profiles according to the different Brazilian regions and the cities of Santos and Itajaí

Region or city	Sample ID	Subtype	NRTI	NNRTI	PI
N	MA012_RC	B		V106A	
	MA013_RC	B			D30N, M46I
	MA027_RC	F	M41L, T215E		D30N, N88D
	MA029_RC	B	M184V		
NE	BA001_RC	B			I54T
	BA004_RC	B	M41L	L100I, K103N	
	BA006_RC	B	M41L, D67N, L210W, T215D		
	BA008_RC	B	M41L, T215CS		M46I, L90M
	BA012_RC	B	K219N		
	BA048_2	B			D30N, M46I
	BA054	F		K103N	
	BA057	B	T215S		
MW	DF006_RC	B	T215S		
	DF012	B	T215D	Y181C	
	DF013_RC	B	T215S		
	DF016	B			G73S
	DF021	B	M41L, L210W, T215D		L24I, M46L, V82A
SE	RJ005_RC	B		K103N	
	RJ018	B	D67G		
	RJ019	B	D67N		
	RJ024_RC	B	V75M, F77L	P225H	
	RJ036_RC	B	M41L, L210W, T215S	Y188L	
	RJ052_RC	B		K101E, K103N, G190A	
S	POA_016	C	M41L, L210W	K101E, V106M, G190A	
	POA_023	C	D67N		
	POA_025	CRF 31_BC			L76V
	POA_052	C			G73S
	POA_053	B			M46I
	POA_054	CRF 31_BC		K103N	
ST	STOS_005	BD		K103N	
	STOS_026	BF	T69D		
	STOS_027	BF	T69D		
	STOS_047	BF		K103N	I54T
	STOS_683	F		Y181I	
	STOS_902	B		K103N	
	ST623	BF	M41L, M184V, L210W, T215Y	Y181I	V32I, M46I, I47V, F53L, I85V
	ST624	B	M41L, D67N, K70R, L74I, M184V, T215F, K219Q	K103N, Y188L	
ITA	ITA_018	C	K65R		
	ITA_107	C	M184I		G73S

N=North, NE=Northeast, MW=Midwest, SE=Southeast, S=South, ST=Santos, ITA=Itajaí.

The overall mean and median CD4+ T cell counts were 253 and 269 cells/µL, respectively, with a range from 3 to 861 cells/µL. The mean and median CD4+ T cell counts among individuals with TDR were 219 and 250 cells/µL, respectively, compared to 257 and 271 cells/µL, respectively, among individuals harbouring wild-type HIV strains, with no significant difference between the groups. The overall mean and median viral loads were 4.68 and 4.70 log_10_ copies/mL, respectively, with a range from 2.0 to 6.34 log_10_ copies/mL. Again, no significant differences were noted in the mean and median viral loads between individuals with or without TDR, which were, respectively, 4.63 and 4.61 log_10_ copies/mL for individuals harbouring TDR strains compared to 4.69 and 4.72 log_10_ copies/mL among individuals harbouring wild-type strains. Regional differences in the CD4+ T cell counts and viral loads are presented in Table 1.

**Figure 1:**
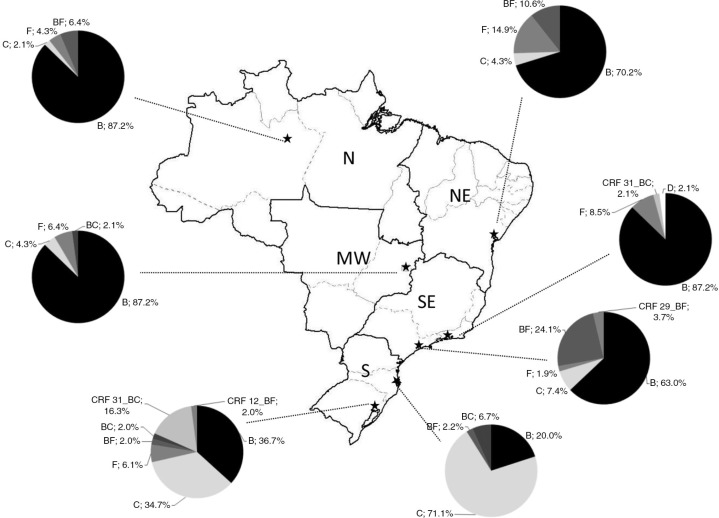
Map of Brazil depicting the different regions and cities in which the survey was conducted and the HIV-1 subtype prevalences.

According to analyses of the protease and RT regions of the *pol* gene, a variety of different subtypes and recombinant forms were detected. Overall, 64.6% of individuals were infected with pure subtype B, 17.3% with subtype C, 6.0% with subtype F, 6.8% with BF recombinants, 1.5% with BC recombinants, 2.7% with CRF31_BC, 0.6% with CRF29_BF, 0.3% with CRF12_BF and 0.3% with subtype D. The regional prevalences of HIV-1 subtypes are shown in Figure 1.

A phylogenetic analysis using a neighbour-joining phylogenetic reconstruction and excluding resistance-related codons revealed no clustering of samples with TDR sequences when genetic distances <1.5% were used to define clusters (data on file).

## Discussion

We identified variable prevalences of TDR, from intermediate (5–15%) to high (>15%), according to geographic region in individuals with HIV that progressed immediately before initiation of ART treatment as prescribed by the attending physician. This report describes a geographically diverse TDR survey that draws upon multiple areas of Brazil, being truly representative of all of the various Brazilian macro-regions. To perform this study, one very populated city was chosen to represent each Brazilian macro-region. Additionally, two other cities, Itajaí and Santos, were included due to the explosive nature of their epidemics, which have been characterized by the high incidence of AIDS and the high number of new HIV cases reported in the last few years (www.aids.gov.br). Evaluation of in-house genotyping assay using DBS in the WHO global laboratory network has previously shown that the reproducibility and accuracy of nucleotide sequence determination and resistance-associated mutation identification from DBS were similar to those previously determined for plasma [16]. In the current study, genotype results have been successfully obtained using DBS for regions located far from a central laboratory able to provide molecular-based testing, including places such as Manaus, which is located in the Brazilian rain forest and has high humidity and temperatures. In our study, the distances from the collecting sites to the central laboratory varied from 74 to 3876 km, with a mean distance of 2510 km, confirming that DBS can provide access to molecular-based testing in spite of large distances and extreme conditions in developing sets. Notably, this study was designed to detect TDR in a group of individuals in whom HIV disease had progressed and who, therefore, were well beyond the HIV transmission event. The overall mean CD4+ T cell count in this group of individuals was 206.5 cells/µL; based on the natural history of HIV progression, it is estimated that the time to achieve such a CD4+ T cell count is an average of eight years [17]. Despite this length of time, a considerable prevalence of TDR was detected.

Previous surveys in Brazil that included several different cities with individuals with recent HIV diagnoses or recent HIV infections demonstrate that the TDR prevalence is generally lower than the prevalence in the present study [3–5, 11, 18–42]. However, we were able to confirm the previously reported high prevalence of TDR in the city of Salvador, located in the Northwest region, and Santos in the Southeast region of Brazil [26].

There is an interesting general trend towards detecting more TDR mutations to NRTI than to NNRTI in individuals with longstanding infections, in contrast to individuals with recent HIV infections [4, 43]. The current study indeed confirmed this trend: the prevalence of resistance was 6.9% to NRTIs and 4.9% to NNRTIs. Twenty-three individuals harboured viruses with NRTI mutations, 16 individuals harboured viruses with thymidine analogue mutations (TAMs) and 8 individuals harboured viruses with other nucleoside analogue mutations (NAMs). Ten individuals harboured viruses with a mutation at RT codon 215, and, as expected from a population harbouring HIV for a long period of time, 8 individuals were infected with so-called revertants (215D/C/S/E, Table 1), which are products of the evolution of T215Y or T215F. Although the revertants themselves do not present any level of phenotypic resistance [44], it is plausible that individuals harbouring these revertants have also acquired the T215Y or T215F strains, which are associated with virologic antiretroviral failure [45]. Of course, it is theoretically possible that the revertants were transmitted rather than the original T215Y or T215F strains; next-generation sequencing techniques may be able to answer this important and interesting question. Furthermore, in accordance with other studies, only three patients in this study presented mutations in the M184 RT codon. Considering that the M184V mutation has a much higher prevalence among individuals with secondary resistance, it is possible that regular genotyping for determining TDR might underestimate the prevalence of this mutation. Although TDR mutations tend to persist over time, M184V might wane more rapidly [6–8]. It is also possible that the APOBEC-related hypermutation plays a role in this process, as valine is encoded by the codon GTG and methionine is encoded by ATG. Interestingly, other resistance mutations besides M184V/I that also cause high fitness cost, such as K65R in one case and D30N in three cases, have also been detected in this survey. The D30N mutation is exclusively selected by Nelfinavir among individuals infected by subtype B viruses, and although this drug has not been used in Brazil since 2007, it has been extensively used in Brazil since 1998.

NNRTI resistance mutations were detected in 16 individuals: 9 harboured viruses with the K103M mutation, and three individuals carried viruses with a mutation at codon 181. Two NNRTI pathways for resistance may exist, depending on the drug used. The use of efavirenz primarily selects for the K103N mutation, which is generally accompanied by the L100I and P225H mutations, whereas nevirapina predominantly selects for the Y181C mutation, which is generally accompanied by the K101E and G190A mutations. The G190A mutation contributes cross-resistance to the second-generation NNRTI etravirine [46].

Another important finding of this study is that almost one-third of individuals harbouring TDR exhibited resistance to two antiretroviral classes, which has been a rare finding in other studies. Furthermore, one individual harboured TDR viruses to three antiretroviral classes. A case-control study performed in the city of Santos, Brazil, previously showed an association of multi-antiretroviral class resistance with virology failure, thus emphasizing the importance of detecting such cases.

This study was able to detect a number of rare HIV subtypes and recombinants. However, it is clear that subtype B still prevails in Brazil, with interesting trends towards higher prevalences of subtype C towards the South and subtypes F and BF towards the North. This study did not reveal differential TDR prevalences according to subtype, as previously suggested. For instance, among recently diagnosed individuals in the South of Brazil, a higher prevalence of TDR among individuals with subtype B than among individuals with subtype C has been reported (Porto Alegre) [47]. As subtypes other than B tend to fix and expand in the Western world, perhaps more attention should be given to antiretroviral resistance vis-à-vis non-B subtypes. One study analysing phenotypic resistance in a limited number of samples from antiretroviral-naïve individuals in Brazil revealed that genotypic correlates of subtype C resistance might not yet be clearly defined [48]. Along a similar line of reasoning, certain phenotypes of natural resistance to PIs might be encountered in subtype F strains [49].

We recognize that the sampling technique used herein might omit important information about TDR in a country of continental size such as Brazil. However, we believe that the results presented here justify checking TDR prior to starting ART in Brazil, even if the samples are collected long after the HIV transmission event, given that almost one-third of patients harboured multi-class drug-resistant HIV genotypes. Furthermore, the use of DBS for antiretroviral resistance monitoring was efficacious and might be cost-effective, especially in settings in which resources are limited and when samples need to be collected in remote regions for later processing in a central laboratory. However, a portion of the samples (9.3%) failed to provide reliable results using this strategy.
